# Single-cell RNA sequencing highlights the influence of innate and adaptive immune response mechanisms in psoriatic arthritis

**DOI:** 10.3389/fimmu.2024.1490051

**Published:** 2025-02-27

**Authors:** Melanie R. Nielsen, Marie Skougaard, Clara Drachmann, Zara R. Stisen, Sisse B. Ditlev, Leon E. Jessen, Lars Erik Kristensen

**Affiliations:** ^1^ Department of Health Technology, Section for Bioinformatics, Technical University of Denmark, (DTU), Kgs., Lyngby, Denmark; ^2^ Department of Clinical Immunology, Aarhus University Hospital, Aarhus, Denmark; ^3^ The Parker Insitute, Copenhagen University Hospital – Bispebjerg and Frederiksberg, Copenhagen, Denmark; ^4^ Copenhagen Center for Translational Research, Copenhagen University Hospital - Bispebjerg and Frederiksberg, Copenhagen, Denmark; ^5^ Department of Clinical Medicine, Faculty of Health and Medical Sciences, University of Copenhagen, Copenhagen, Denmark

**Keywords:** psoriatic arthritis, psoriasis, scRNAseq, transcriptomics, bioinformatics, inflammation, differential expression analysis, gene set enrichment analysis

## Abstract

**Introduction:**

Psoriatic arthritis (PsA) is a chronic immune-mediated inflammatory disease displaying heterogeneous symptoms. However, the association between the clinical heterogeneity of PsA and disease immunopathogenesis remains poorly understood complicating diagnostic precision. A knowledge gap remains on whether it is possible to distinguish the clinical PsA phenotypes on the immune cellular level. The primary aim of the study was to explore the differences in gene expression profiles comparing PsA patients without cutaneous psoriasis (PsA-only) and PsA patients with cutaneous psoriasis (PsA/PsC). The secondary aim was to describe the transcriptional patterns in PsA patients compared with healthy controls.

**Methods:**

The study applied single-cell RNA sequencing (scRNAseq) using the BD Rhapsody™ Single-Cell Analysis System to evaluate peripheral blood mononuclear cells (PBMCs) from 70 PsA patients and 10 healthy controls. Differential expression (DE) analysis and gene set enrichment analysis (GSEA) were applied to evaluate differentially expressed genes (DEGs) and enriched signaling pathways, respectively.

**Results:**

The DE analysis and GSEA comparing PsA-only and PsA/PsC patients with healthy controls, respectively, revealed divergent results involving both innate and adaptive immune mechanisms, which might be associated with differences in the clinical phenotype. No DEGs were discovered in the direct comparison of PsA-only and PsA/PsC patients.

**Discussion:**

The single-cell transcriptome profiling provided insight into the heterogeneity of PsA patients as the discovered DEGs and the GSEA did demonstrate differences in signaling associated with inflammation comparing PsA patients with and without cutaneous psoriasis.

## 1 Introduction

Psoriatic arthritis (PsA) is a chronic immune-mediated inflammatory disease driven by a dysregulation of the immune response leading to a heterogeneous array of clinical manifestations, including one or more of the following: joint pain and swelling, spinal disease, cutaneous psoriasis (PsC), enthesitis, nail abnormalities, and dactylitis (1).

The association between the clinical heterogeneity of PsA and disease immunopathogenesis remains poorly understood, which complicates diagnostic precision as treatment response may vary between individual patients suffering from different symptoms (2). The majority of patients diagnosed with PsA present with preexisting PsC. However, 20% of PsA patients develop PsC after the debut of arthritis (3), whereas 1%–2% of patients will never develop PsC (4). This complicates treatment decision-making and can lead to a prolonged clinical process for patients before the correct diagnosis is established and the medical therapy with the best effect on the inflammatory response and clinical symptoms is provided. Furthermore, it has been estimated that 20%–30% of PsA patients have no effect of existing treatment options (5, 6) and it is well known that medical treatment in some cases can worsen existing symptoms (7). However, the immunological knowledge to support clinical practice in treatment decision-making is lacking. Understanding the underlying immunopathogenesis, including immune cell composition and functionality specific to the various PsA clinical phenotypes, can pave the way for more personalized, targeted, and effective treatment. Existing research has indicated different immune cellular phenotypes to be associated with different clinical phenotypes, including high clinical PsC scores being influenced by natural killer cells and CD8+ T cells (8). Moreover, an evaluation of the genetic predisposition to disease has identified different risk variants comparing PsC subtypes, including PsA (9), implying that diverse immune response mechanisms might be responsible for the observed clinical heterogeneity.

Here, we present a translational study utilizing high-throughput single-cell RNA sequencing (scRNAseq) to explore transcriptional pattern and gene expression profiles of PsA patients to uncover the association between transcriptional characteristics and clinical phenotypic heterogeneity, comparing PsA patients suffering from concurrent PsC (PsA/PsC) and PsA patients without cutaneous psoriasis (PsA-only).

## 2 Materials and methods

### 2.1 Cohort description

#### 2.1.1 Patients

A total of 70 PsA patients, comprising 30 PsA patients planned for initiation of tumor necrosis factor alpha inhibitor (TNFi), 20 PsA patients planned for initiation of interleukin 17A inhibitor (IL-17Ai), and 20 patients planned for initiation of methotrexate (MTX), were included from the Parker Institute’s consecutive PsA patient cohort (PIPA). PIPA inclusion and exclusion criteria are described in the PIPA cohort paper (10). Included patients were further stratified based on cutaneous PsC involvement quantified by the Psoriasis Area Severity Index (PASI), i.e., PsA/PsC with PASI >2 and PsA-only with PASI = 0. Patients with PASI >0 and ≤2 were excluded from the current study (**Supplementary Figure S1**). Peripheral blood mononuclear cells (PBMCs) and clinical data were retrieved from the PIPA (10) baseline visit conducted adjacent to the initiation of medical therapy. Additionally, 10 healthy, age- and gender-matched individuals were included as controls. The project was conducted in accordance with the Declaration of Helsinki with ethical approval obtained from the Danish Ethical Committee of the Capital Region of Denmark (J.no.: H-18024568) and the General Data Protection Regulation approved by the Capital Region of Denmark (J.no.: BFH-2015-043). All patients provided written informed consent before inclusion.

#### 2.1.2 Clinical and patient-reported outcomes

A clinical evaluation was performed by a physician to examine the impact of PsA, including 66/68 joint assessments to evaluate swollen and tender joints, respectively. Furthermore, the Spondyloarthritis Research Consortium of Canada (SPARCC) enthesitis score evaluating 16 sites, an 18-site fibromyalgia tender point count, and Psoriasis Area Severity Index (PASI) ranging from 0 to 72 were obtained from the clinical examination. Patient-reported outcome included the Visual Analogue Scale (VAS) depicting patient global health, pain and fatigue ranging from 0 mm to 100 mm, Psoriatic Arthritis Impact of Disease (PSAID) ranging from 0 to 10, and Health Assessment Questionnaire – Disability Index (HAQ-DI) ranging from 0 to 3. Composite measures, including Disease Activity in PsA (DAPSA) (11) and Disease Activity Score (DAS28CRP) (12), were calculated to quantify PsA disease activity.

### 2.2 Experimental workflow

#### 2.2.1 Separation of PBMCs

Peripheral blood was collected in EDTA vacutainer tubes (Greiner Bio-One, Kremsmünster, Germany) for immediate processing to retrieve PBMCs, which were isolated performing density gradient centrifugation, including Ficoll-Paque solution (GE Healthcare, Uppsala, Sweden). PBMCs were cryopreserved in heat-inactivated fetal bovine serum (FBS) (Gibco, Grand Island, NY USA) with 10% dimethyl sulfoxide (ITW Reagents, Darmstadt, Germany) with initial controlled freezing to −80°C ensured by the CoolCell™ (Corning) cryogenic storage box and subsequently transfer to liquid nitrogen until further analysis.

#### 2.2.2 Single-cell capture and cDNA library preparation

The BD Rhapsody™ Single-Cell Analysis System (BD Biosciences) (13) was implemented for the preparation of cDNA libraries. PBMCs were thawed and washed twice using preheated RPMI 1640 (Gibco) with 10% FBS (Gibco). Cells were stained with Calcein AM (Thermo Fisher Scientific) and Draq7 (BD Biosciences) before evaluation of viability and cell concentration on the BD Rhapsody Scanner. Cells were labeled with the BD^®^ Single-Cell Multiplexing Kit (BD Pharmingen™) (14) using different sample tags 1–12 and washed twice with stain buffer (BD Pharmingen™). Multiplex-labeled cells from two patients were pooled and loaded onto the primed BD Rhapsody™ Cartridge and transferred to the BD Rhapsody Express station (15). Magnetic cell capture beads were loaded to the cartridge followed by an incubation step to ensure single-cell capture in the microwells of the cartridge, and a washing step to remove excessive beads. Cells captured by the magnetic beads were lysed within the microwells and beads were recovered before reverse transcription. The median retrieval rate of viable cells per patient was 9,946 [IQR 8,726; 11,332] with a median cell viability of 95.3% [IQR 90.4%; 97.9%] and a median doublet rate of 5.1% [IQR 4.6%; 5.9%]. The mRNA whole-transcriptome approach (WTA) (16) was implemented for priming, extension, and amplification of transcripts. Library indexes were added through polymerase chain reaction (PCR) generating the final cDNA libraries and sample tag libraries, separately. All procedures were performed strictly in accordance with the manufacturer’s protocol.

#### 2.2.3 Sequencing

WTA cDNA libraries and sample tag libraries, including relevant cell labels and unique molecular identifiers (UMI), from five to six cartridges (10–12 patient samples) were pooled before sequencing. Pooled libraries were spiked with 20% PhiX and sequenced (paired-end, 2 × 150 bp) on the NovaSeq 6000 S4 (Illumina). Sequencing was conducted at the Kennedy Center, Department of Clinical Genetics, Centre of Diagnostic Investigation, Rigshospitalet, Copenhagen, Denmark.

### 2.3 Statistical and single-cell data analyses

An overview of the steps involved in the single-cell RNA-seq analysis pipeline is provided in **Supplementary Figure S2**. Baseline characteristics were presented as numbers with percentages for categorical variables and median with interquartile ranges (IQR) for continuous variables. Differences between groups in baseline demographics and clinical characteristics were evaluated with chi-squared test for categorical variables and one-way ANOVA for continuous variables, utilizing the “tableone” package (version 0.13.2) in R.

#### 2.3.1 Quality control, cell annotation, and reference mapping

The BD Rhapsody™ WTA Analysis Pipeline incorporated in the Seven Bridges Genomics platform was used for processing of the raw fastq-files to generate gene count matrices (17). This involved filtering by read quality, aligning reads to the GRCh38 human reference genome, correcting for UMI errors using recursive substitution error correction (RSEC) and sample demultiplexing. The raw unfiltered gene count matrices were further processed by CellBender v0.3.0 (18) to eliminate technical artifacts by reducing counts due to ambient RNA molecules and random barcode swapping. Subsequent quality control was conducted using the Seurat v5.0.1 R-package (19). Cells with less than 500 UMI counts or 200 distinct gene counts were filtered out. To accommodate the varying proportions of mitochondrial content across samples, sample-dependent thresholds were selected based on the 95th percentile of mitochondrial content within each sample’s cells. For each sample, the 95th percentile was rounded up to the nearest value of 20%, 25%, 30%, or 35%, and cells with higher mitochondrial proportions were filtered out. Eight samples were excluded from the analysis as they did not pass quality control due to much higher mitochondrial content and fewer gene counts than the majority and were therefore suspected to be compromised and four samples were excluded due to technical issues. Additionally, cells with a hemoglobin (HBA1, HBA2, HBB, HBD, HBM, HBG1, HBZ, HBQ1) expression greater than 0.1% were also filtered out. While the sample determination algorithm provided by BD Biosciences removes between-sample doublets, the DoubletFinder R-package V2.0.3 (20) was used for the removal of within-sample doublets using an expected proportion of doublets of 2%.

The count matrix of each sample was individually normalized using SCTransform (19), which identified 3,000 variably expressed genes per sample. The Azimuth anchor-based reference mapping framework was applied to map each sample to a PBMC CITE-seq reference of 161,764 cells of annotated cell types offered by Seurat (19). Anchors between each sample and the reference were identified using the “FindTransferAnchors” function with 50 dimensions and a pre-computed reference dimensional reduction based on supervised PCA (SPCA). Next, these anchors were supplied to the MapQuery function to map each cell of a given query sample to one of the reference cell types. The cell type annotation process was evaluated by considering the highly expressed genes of each predicted cell type as well as the prediction scores derived from Azimuth. Having verified the annotation, all query samples were merged, and cells predicted doublets were removed. Finally, to check for novel populations in the query data, we merged all reference and query cells, and a new Uniform Manifold Approximation and Projection (UMAP) embedding was computed. It was further investigated whether samples could be clustered by a PCA on the sample level, using the fractions of each cell type as input. In addition, a batch-dependent gene ambient metric was computed. This metric was calculated by aggregating counts for each gene found in barcodes with fewer than 100 total gene counts, which indicate non-viable cells. This metric was utilized in subsequent analyses to determine whether a gene’s expression difference stemmed from contamination with ambient RNA or biological factors.

#### 2.3.2 Differential expression analysis

Differentially expressed genes (DEGs) were analyzed at baseline between **A)** PsA/PsC patients and PsA-only patients, **B)** PsA/PsC patients and healthy controls, **C)** PsA-only patients and healthy controls, and **D)** all PsA patients and healthy controls. For all differential expression (DE) analyses, an initial quality control step was conducted based on the subset of data involved in the analysis. This involved re-clustering of the data, to ensure that all samples were represented in each cluster, as well as visual inspection of the generated UMAPs. Next, pseudo-bulks were produced by aggregating the raw counts per cell type and sample combination. Sample-cell type combinations with zero counts and genes observed in less than five cells from such a combination were removed before DE analysis. For consensus, all analyses were performed on each identified cell type separately using the three tools DESeq2 (21), EdgeR (22), and Limma-voom (23). Venn diagrams were generated to visualize the overlapping genes across tools (**Supplementary Figure S3**). Only genes found to have an absolute log2 fold change > 0.5 and found to be significantly differentially expressed (adjusted p-value < 0.05) by at least two of the tools or found to have an adjusted p-value of < 0.01 by a single tool were considered significantly differentially expressed. The 75th percentile was used for filtering based on ambient gene count.

#### 2.3.3 Gene set enrichment analysis

The ClusterProfiler R package V 4.10.0 (24) was applied to perform gene set enrichment analysis (GSEA), using genome-wide annotation for the human reference dataset available from the R package *org.Hs.eg.db* (25). The analysis was conducted in cases where more than 100 genes were found significantly differentially expressed per model setup. The input for the analysis included all the identified differentially expressed genes. To rank the genes, for the GSEA we performed a principal component analysis (PCA) on the log2 fold changes obtained from DESeq2, EdgeR, and Limma-voom and used the first principal component (PC) for gene ranking (**Supplementary Figure S4**). GSEA was performed on all genes for each cell type. Pathways with an adjusted p-value < 0.05 were considered significant and retained for further analysis.

## 3 Results

This study utilized scRNAseq to explore transcriptional pattern and gene expression profiles of PsA patients, examining the association between transcriptional characteristics and clinical phenotypic heterogeneity, comparing PsA/PsC and PsA-only. A total of 39 PsA patients, including 19 PsA/PsC patients and 20 PsA-only patients, were included for the primary analysis exploring the differences in transcriptional patterns of PsA patients with different clinical phenotypes defined by cutaneous disease involvement. A total of 58 PsA patients and 10 healthy controls were included to explore the difference between PsA patients and healthy controls (**Supplementary Figure S1**). No statistically significant differences were found at baseline comparing PsA/PsC (PASI 4.80 [IQR 3.15; 5.85]) and PsA-only (PASI 0.00 [IQR 0.00; 0.00]) patients with regard to age, disease duration, type of biological disease-modifying anti-rheumatic drugs (bDMARDs), number of previous bDMARDs, and clinical outcome, including SPARCC, tender point count, DAPSA, and DAS28CRP (**Table 1**). Statistically significant differences between the groups were found comparing sex, VAS patient global health, and VAS patient fatigue with PsA-only patients scoring higher than PsA/PsC patients. This trend was indicated in measuring VAS patient pain as well, but the result did not reach statistical significance.

**Table 1:** Baseline characteristics.

	All PsA (n=58)	PsA-only (n=20)	PsA/PsC (n=19)	p value †	HCs (n=10)	p value ‡
Age	52.57 (12.33)	52.94 (14.12)	48.29 (12.16)	0.279	46.77 (10.42)	0.166
Female	26 (44.8)	5 (25.0)	12 (63.2)	**0.038**	5 (50.0)	1.000
Disease duration	8.44 (10.08)	8.75 (12.72)	7.07 (7.55)	0.620		
Active treatment				0.116		
- TNFi	17 (29.3)	8 (40.0)	4 (21.1)			
- IL-17Ai	16 (27.6)	6 (30.0)	3 (15.8)			
- MTX	25 (43.1)	6 (30.0)	12 (63.2)			
Previous bDMARDs				0.265		
- 0	8 (13.8)	3 (15.0)	1 (5.3)			
- 1	30 (51.7)	8 (40.0)	11 (57.9)			
- 2	12 (20.7)	4 (20.0)	5 (26.3)			
- ≥3	8 (13.8)	5 (25.0)	2 (10.6)			
SPARCC enthesitis	3.00 [1.00, 5.00]	4.50 [1.75, 5.00]	3.00 [1.00, 6.00]	0.713		
Tender point count	1.00 [0.00, 6.00]	4.00 [0.00, 8.25]	0.00 [0.00, 3.50]	0.160		
PASI	1.20 [0.00, 3.00]	0.00 [0.00, 0.00]	4.80 [3.15, 5.85]	**<0.001**		
DAPSA	29.80 [22.00, 42.50]	32.60 [24.47, 40.47]	29.00 [20.05, 45.40]	0.844		
DAS28CRP	4.14 [3.72, 4.92]	4.40 [3.86, 4.96]	4.09 [3.73, 4.90]	0.613		
VAS pt. Global	69.00 [50.00, 79.00]	76.00 [68.25, 91.25]	57.00 [33.50, 74.00]	**0.012**		
VAS pt. Pain	64.00 [30.00, 79.00]	70.50 [56.75, 85.75]	41.00 [23.00, 77.50]	0.064		
VAS pt. Fatigue	67.00 [50.00, 82.00]	79.50 [67.75, 89.25]	60.00 [37.50, 77.00]	**0.038**		
PSAID	5.68 [3.17, 6.54]	6.09 [4.78, 7.63]	4.81 [2.58, 6.52]	0.164		
HAQ	1.00 [0.50, 1.63]	1.13 [0.60, 1.75]	0.88 [0.44, 1.19]	0.190		

Baseline characteristics presented as number with corresponding percentages for categorical variables and median with interquartile ranges (IQR) for continuous variables. P values (†) represent the differences between PsA/PsC and PsA-only patients. P-values (‡) represent the difference between all PsA patients and healthy controls. HCs, healthy controls; TNFi, tumor necrosis factor alpha inhibitor; IL-17Ai, interleukin 17A inhibitor; MTX, methotrexate; bDMARDs, biological disease modifying anti-rheumatic drugs; SPARCC, Spondyloarthritis Research Consortium of Canada; PASI; Psoriasis Area Severity Index; DAPSA, Disease Activity in Psoriatic Arthritis; DAS28CRP, Disease Activity Score; VAS, Visual Analogue Scale; PSAID, Psoriatic Arthritis Impact of Disease; HAQ, Health Assessment Questionnaire.Bold text indicates statically significant p-values.

### 3.1 Cell type abundance profiles were similar in PsA patients

**Figure 1:**
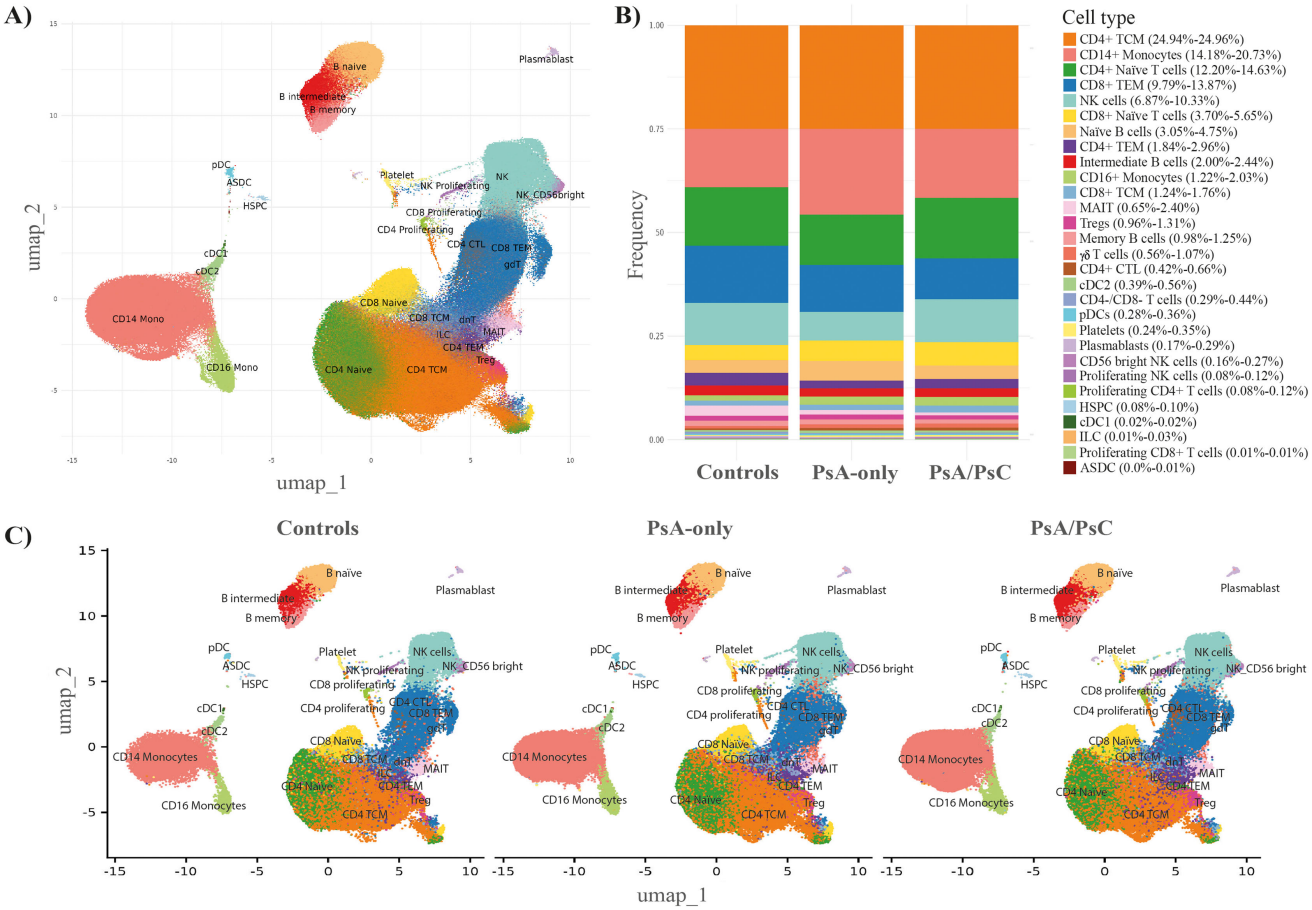
Immune cell abundance profiles. Cell type annotation of the data **(A)** UMAP embedding of the entire dataset labeled by referenced-based cell type annotation revealed 27 unique cell type clusters identified using all 68 samples. **(B)** Stacked bar chart including the fraction of each annotated cell type, in PsA-only, PsA/PsC, and healthy controls (49 samples). **(C)** UMAP embeddings split by PsA-only, PsA/PsC patients, and healthy controls (49 samples). PsA-only, psoriatic arthritis without cutaneous psoriasis; PsA/PsC, psoriatic arthritis with cutaneous psoriasis; UMAP, uniform manifold approximation and projection; PsA, psoriatic arthritis; PsC, cutaneous psoriasis; CD, cluster of differentiation; TCM, central memory T cells; TEM, effector memory T cells; NK, natural killer; MAIT, mucosal associated invariant T cells; Tregs, regulatory T cells; cDC2, conventional dendritic cells 2 (CD1c-positive); pDCs, plasmacytoid dendritic cells; HSPC, hematopoietic stem and progenitor cells; cDC1, conventional dendritic cells 1 (CD141-positive); ILC, innate lymphoid cells; ASDC, AXL+ dendritic cells; CTL, cytotoxic T cells.

**Figure 2:**
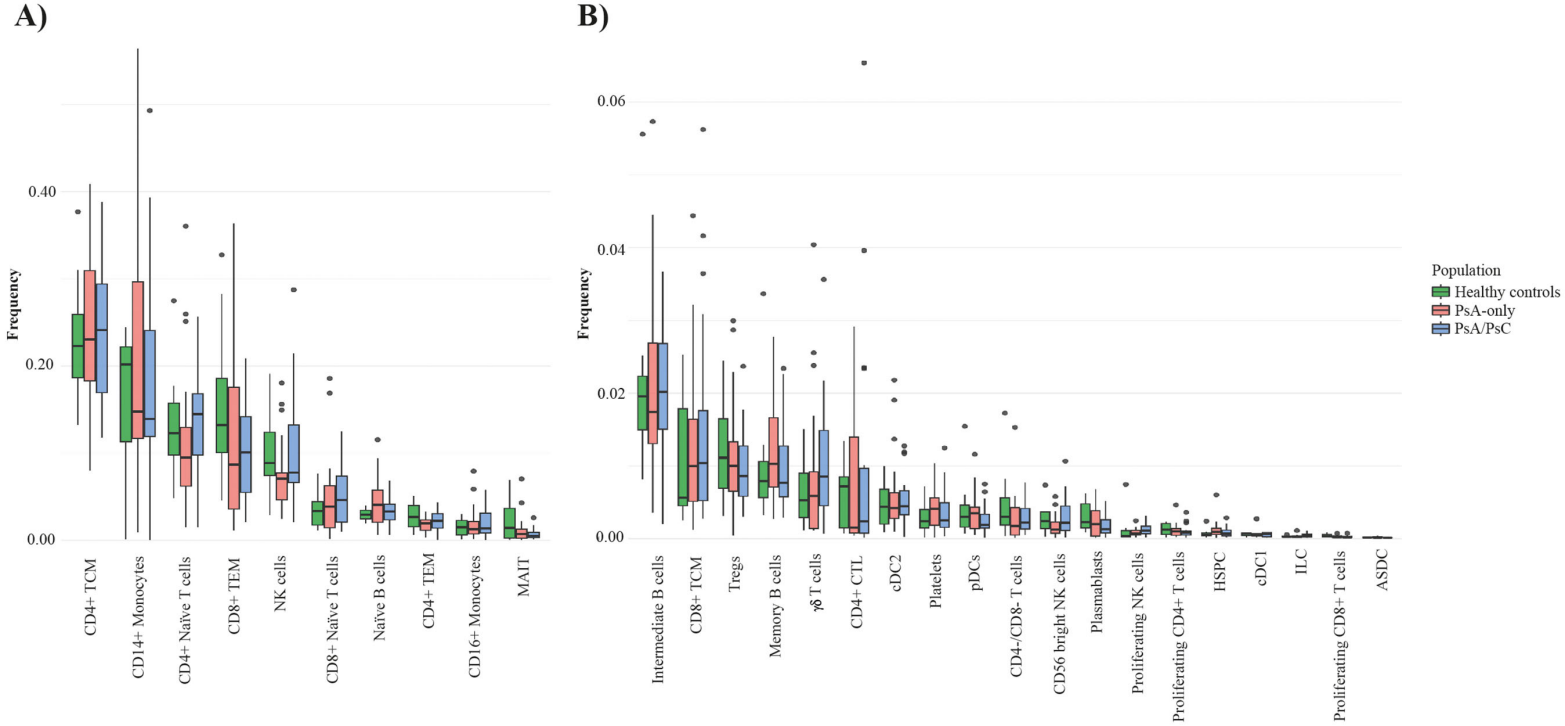
Cell type frequencies in PsA-only patients, PsA/PsC patients, and healthy controls. Boxplot of the cell type frequency distribution, colored by the groups PsA-only (red), PsA/PsC (blue), and healthy controls (green); 49 samples in total. **(A)** Cell types with a frequency above 1%. **(B)** Cell types with a frequency lower than 1%. PsA, psoriatic arthritis; PsA/PsC psoriatic arthritis with cutaneous involvement; PsA, psoriatic arthritis; PsC, cutaneous psoriatic; CD, cluster of differentiation; TCM, central memory T cells; TEM, effector memory T cells; NK, natural killer; MAIT, mucosal associated invariant T cells; Tregs, regulatory T cells; CTL, cytotoxic T cells; conventional dendritic cells 2 (CD1c-positive); pDCs, plasmacytoid dendritic cells; HSPC, hematopoietic stem and progenitor cells; cDC1, conventional dendritic cells 1 (CD141-positive); ILC, innate lymphoid cells; ASDC, AXL+ dendritic cells; CTL, cytotoxic T cells.

A total of 469,438 cells from 68 patient samples (58 PsA patients and 10 healthy controls) were retained after quality control and included for further analysis. There were 27 distinct immune cell types identified by the cell type annotation and clustered according to the identified cell types (**Figure 1A**) with no discovery of novel cell populations (**Supplementary Figure S5**). The abundance of the different cell types was overall consistent across all samples (**Supplementary Figure S6**) and across the three examined groups, i.e., PsA/PsC (148,078 cells), PsA-only (142,563 cells), and healthy controls (58,138 cells), with a similar distribution of cell type clusters (**Figure 1C**). The overall cell type distribution and frequency within the examined groups were similar with CD4+ T central memory cells (TCM), CD14+ monocytes, and CD4+ naïve T cells, being the most dominant cell types accounting for 53.0%–57.7% of the examined immune cells of all three groups (**Figure 1B**), although with varying fractions of CD14+ monocytes, and a lower fraction of NK cells in PsA-only patients (**Figures 2A, B**). The PCA using cell type fractions did not reveal any distinct clustering of the patients, with 74.29% variance explained by the first two PCs (**Supplementary Figure S7**).

### 3.2 Differential expression analysis comparing PsA-only and PsA/PsC patients

The examination of DEGs comparing PsA-only patients and PsA/PsC patients was conducted, aiming to resolve the diversity and heterogeneity of clinical PsA phenotypes. No differentially expressed genes were found comparing the two patient groups.

### 3.3 Identification of differentially expressed genes in PsA compared with healthy controls

**Figure 3:**
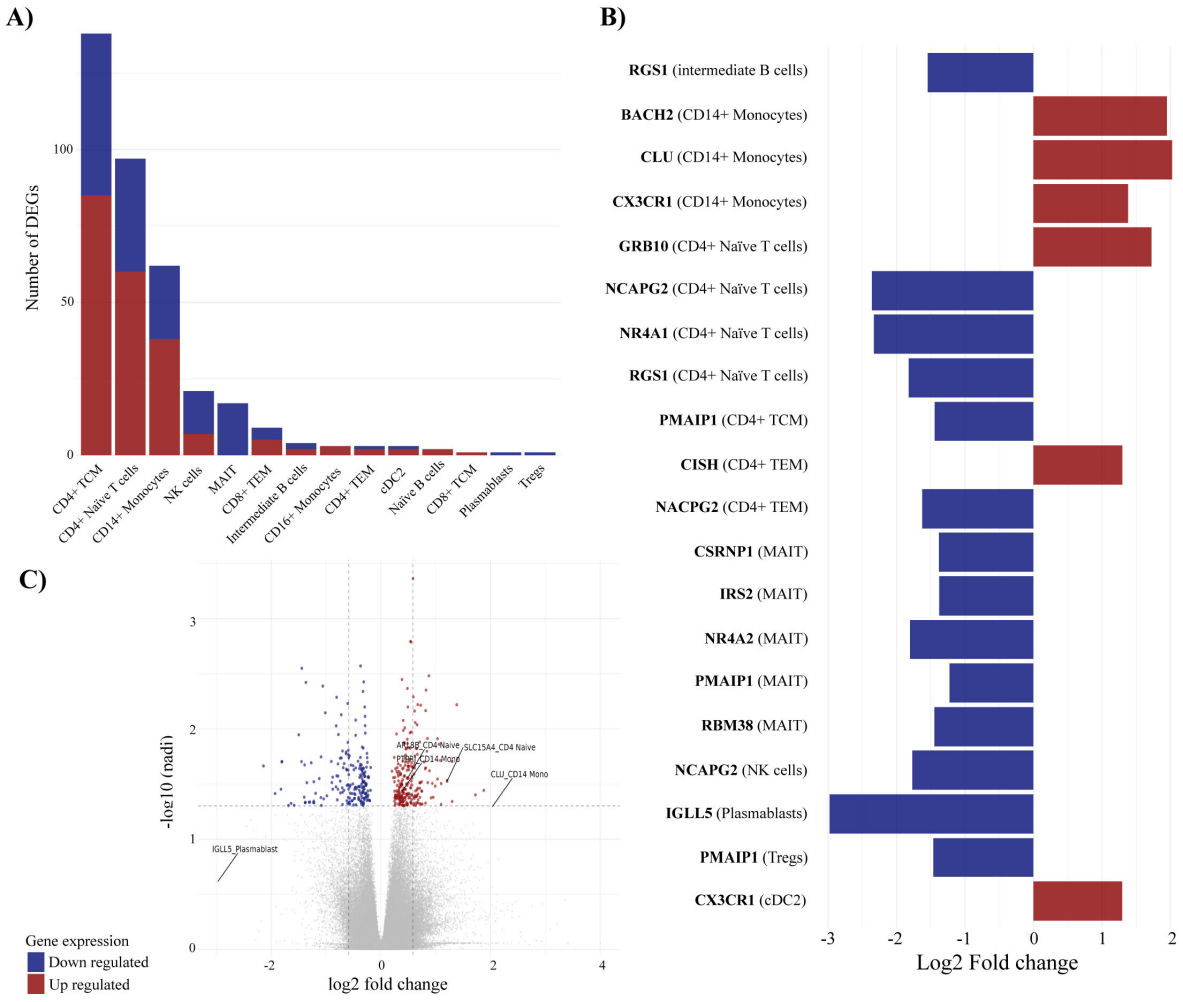
Differentially expressed genes (DEGs) associated with PsA compared with healthy controls. Differential expression analysis revealed 298 unique differentially expressed genes comparing PsA patients and healthy controls. **(A)** visualizes the number of DEGs in individual immune cell types, **(B)** displays the top-20 DEGs and the cell association, and **(C)** is the volcano plot defining the DEGs with p-adjusted cutoff at 0.05 and log2 fold change cutoff at 0.5 representing the distribution of non-DEGs and DEGs. The mean values for p-adjusted and log2 fold change across all three tools (DESeq2, edge R, and Limma-voom) are used for the visualization in **(B, C)**. For all figures, it applies that blue corresponds to downregulated genes, whereas red corresponds to upregulated genes. TCM, central memory T cells; NK, natural killer; MAIT, mucosal associated invariant T cells; TEM, effector memory T cells; cDC2, conventional dendritic cells 2 (CD1c-positive); Tregs, regulatory T cells.

A comparison between all PsA patients (n=58) and healthy controls (n=10) revealed a total of 298 unique significant DEGs (**Supplementary Table S1**), which were mainly identified within CD4+ TCM, CD4+ naive, CD14+ monocytes, and NK cells (**Figure 3A**). Several unique DEGs (**Figure 3C**) were identified across multiple cell types. Among the top 20 DEGs, by absolute log2 fold change, were genes RGS1, CX3CR1, NCAP2G, and PMAIP1, which were represented in >1 cell type (**Figure 3B**). RGS1 (*regulator of G-protein signaling 1*) was downregulated in both intermediate B cells and CD4+ naïve T cells, CX3CR1 (*C-X3-C Motif Chemokine Receptor 1)* was found upregulated in CD14+ monocytes and cDC2s, and NCAPG2 (*Non-SMC Condensin II Complex Subunit G2*), a protein-encoding gene important to cell division, was downregulated in CD4+ naive T cells, CD4+ TEM, and NK cells. Additionally, PMAIP1 (*Phorbol-12-Myristate-13-Acetate-Induced Protein 1*), a pro-inflammatory and pro-apoptotic gene, was found downregulated in CD4+ TCM, MAIT cells, and Tregs. Out of the top 20 DEGs, 13 were associated with T-cell subtypes (**Figure 3B**), demonstrating the well-known importance of T cells in PsA immunopathogenesis. Interestingly, five of the top 20 DEGs were found downregulated in MAIT cells, namely, CSRNP1, IRS, NR4A2, PMAIP1, and RBM38. CISH and CLU were found upregulated in I) CD4+ TCM and CD4+ TEM and II) CD14+ monocytes, respectively. Several genes were found differentially expressed in multiple cell subtypes, including upregulated GIMAP family genes, up- and downregulated ZNFs and TMEMs, and downregulated ZTBTs and PDZD8 (**Supplementary Table S1**).

### 3.4 DEGs associated with PsA-only and PsA/PsC compared with healthy controls

**Figure 4:**
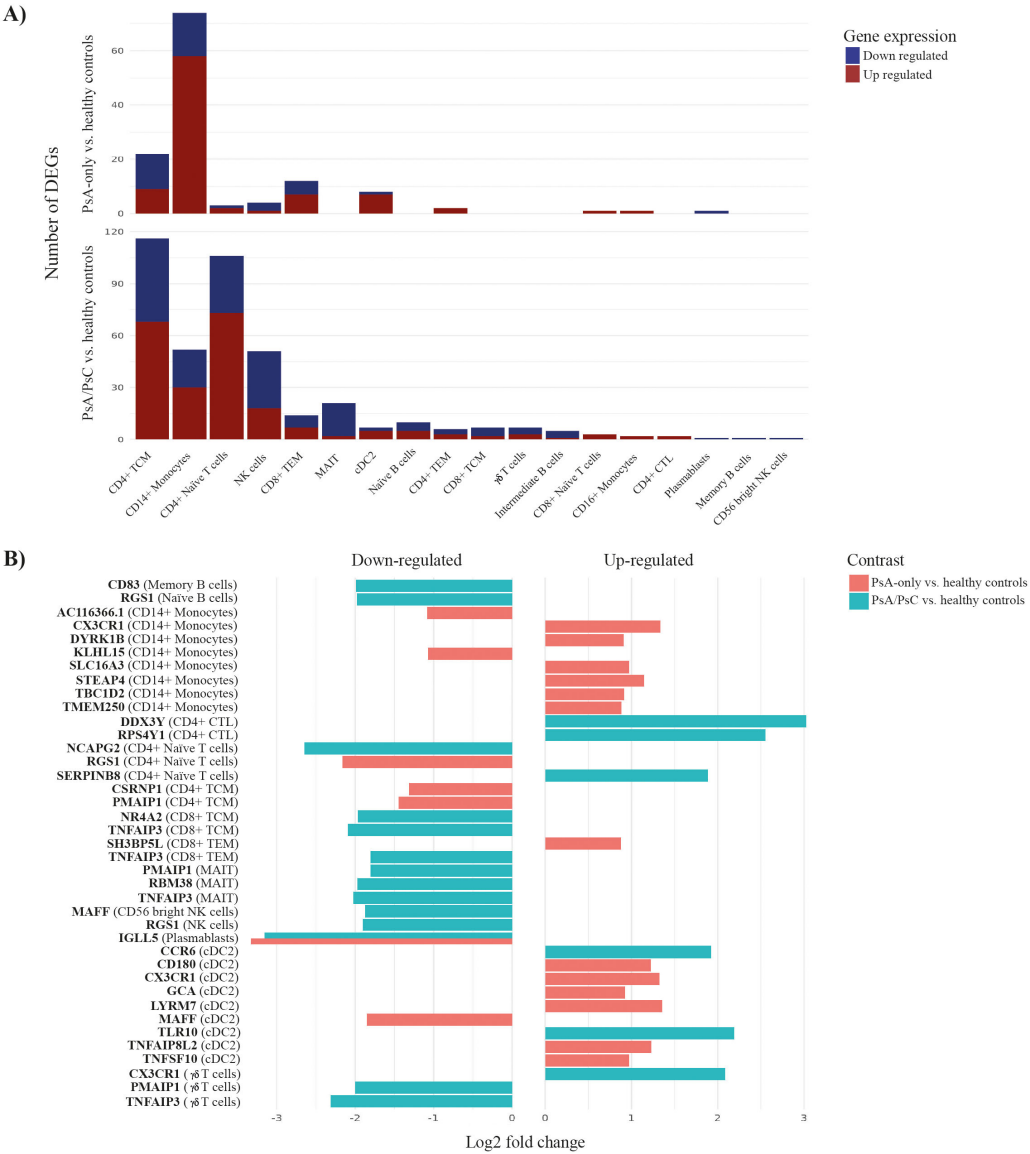
Differentially expressed genes (DEGs) associated with PsA-only and PsA/PsC compared with healthy controls. Differential expression analysis revealed 113 and 308 unique differentially expressed genes between PsA-only patients and PsA/PsC patients compared with healthy controls, respectively. **(A)** visualizes the number of DEGs in individual immune cell types, **(B)** displays the top-39 DEGs and the cell associations. The x-axis log2 fold change represents the mean log2 fold change across all three tools (DESeq2, edgeR, Limma-voom). PsA, psoriatic arthritis; PsC, cutaneous psoriatic; CD, cluster of differentiation; TCM, central memory T cells; TEM, effector memory T cells; NK, natural killer; MAIT, mucosal associated invariant T cells; Tregs, regulatory T cells; CTL, cytotoxic T cells; conventional dendritic cells 2 (CD1c-positive); pDCs, plasmacytoid dendritic cells; HSPC, hematopoietic stem and progenitor cells; cDC1, conventional dendritic cells 1 (CD141-positive); ILC, innate lymphoid cells; ASDC, AXL+ dendritic cells; CTL, cytotoxic T cells.

Grouping PsA patients based on PsC, a total of 113 unique genes were identified as significantly differentially expressed in PsA-only patients compared with healthy controls (**Supplementary Table S2**). On the other hand, 308 unique genes were identified as significantly differentially expressed in PsA/PsC patients compared with healthy controls (**Supplementary Table S3**), respectively (**Supplementary Figure S8**). DEGs were mainly found in CD14+ monocytes for PsA-only patients and CD4+ TCM and CD4+ Naïve T cells for PsA/PsC patients (**Figure 4A**), with an overlap in 49 unique DEGs appearing in both PsA-only and PsA/PsC patients compared with healthy controls. Among the top 20 DEGs based on log2 fold change, only downregulation of IGLL5 in plasmablast cells was observed in both PsA-only and PsA/PsC patients (**Figure 4B**). Overlapping DEGs associated with both PsA-only and PsA/PsC compared with healthy controls, respectively, include inflammation-associated genes such as EOMES and CX3CR1. EOMES was found upregulated in CD8+ TEM in both PsA-only and PsA/PsC, and in MAIT and NK cells in PsA/PsC patients, whereas CX3CR1 was upregulated in CD14+ monocytes and cDC2 in both PsA-only and PsA/PsC patients. Furthermore, in NK and γδ T cells in PsA/PsC patients. Additional overlapping genes included TNFAIP family gene members and TNFAIP8L2 upregulated in cDC2 cells of both patient groups and in CD4+ TCM of PsA/PsC patients, and TNFAIP3 (**Figure 4B**) was downregulated in multiple cell types (CD4+ naïve T cells, CD4+ TCM and TEM, CD8+ TCM and TEM, γδ T cells, and MAIT and NK cells). Several differentially expressed long non-coding (lnc)RNA were exposed in the study, which has been implicated in the development of inflammation and the immunopathogenesis of PsA. However, the exact mechanisms of immune-related lncRNAs in the inflammation response remain largely unknown (26, 27).

### 3.5 Both innate and adaptive molecular mechanisms were associated with PsA

**Figure 5:**
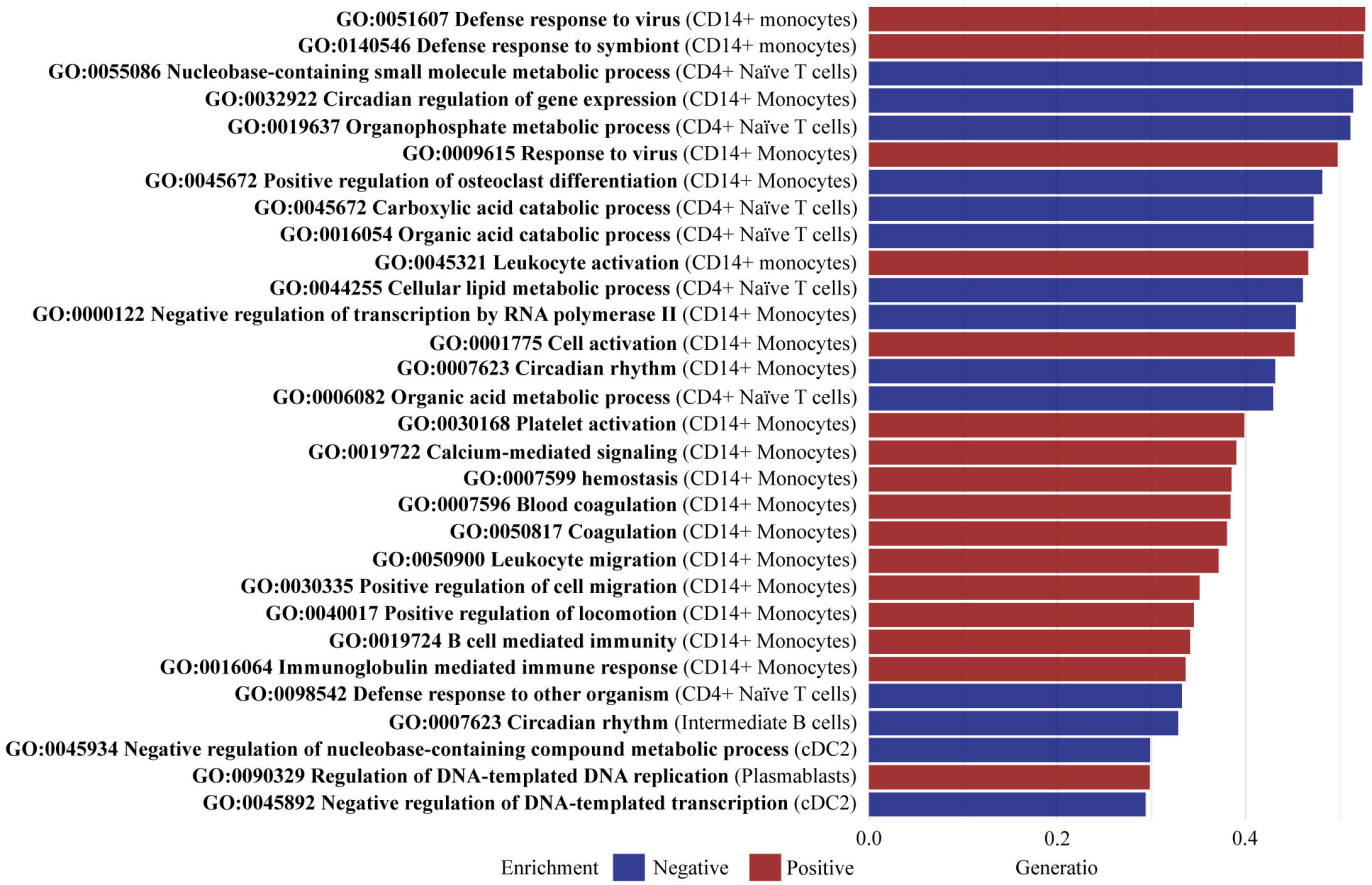
Top 30 enriched pathways defined by gene set ratio. Gene set enrichment analysis identified a total of 128 significantly enriched pathways (adjusted p-value < 0.05) including 49 negatively enriched pathways and 79 positively enriched pathways. The figure visualizes the top 30 pathways with the highest gene set ratio, describing the overlap between the identified DEGs, and the reference list of genes in the pathway. All 128 pathways are reported in **Supplementary Table S1**. CD, cluster of differentiation; cDC2, conventional dendritic cells 2 (CD1c-positive).

GSEA was implemented to identify enriched pathways in all PsA patients compared with healthy controls. Based on the detected DEGs, a total of 128 pathways were found to be significantly enriched (adjusted p-value < 0.05), with 49 negatively enriched and 79 positively enriched pathways (**Supplementary Table 4**). In CD14+ monocytes, pathways such as *positive regulation of osteoclast differentiation* (GO:0045672), *leukocyte activation* (GO:0045321), and *leukocyte migration* (GO:0050900) were enriched having a high gene ratio, spanning from 35% to 48% overlap between the identified DEGs and the list of genes involved in the pathways (**Figure 5**). The significant DEGs CLU and PTPRJ were part of the core enrichment gene set for leukocyte activation and were upregulated in CD14+ monocytes of PsA patients compared with healthy controls. Additionally, the pathway *defense response to other organism* (GO:0098542) was enriched in CD4+ Naive T cells (**Figure 5**). The core enrichment genes included ARL8B and SLC15A4, which were upregulated in PsA patients compared with healthy controls. The pathways *Immunoglobulin mediated immune response* (GO:0016064) and *B cell-mediated immunity* (GO:0019724) were enriched in plasmablast. The significant DEG IGLL5 part of the core enrichment was downregulated in plasmablast cells of PsA patients compared with healthy controls.

### 3.6 GSEA comparing PsA-only and PsA/PsC with healthy controls

**Figure 6:**
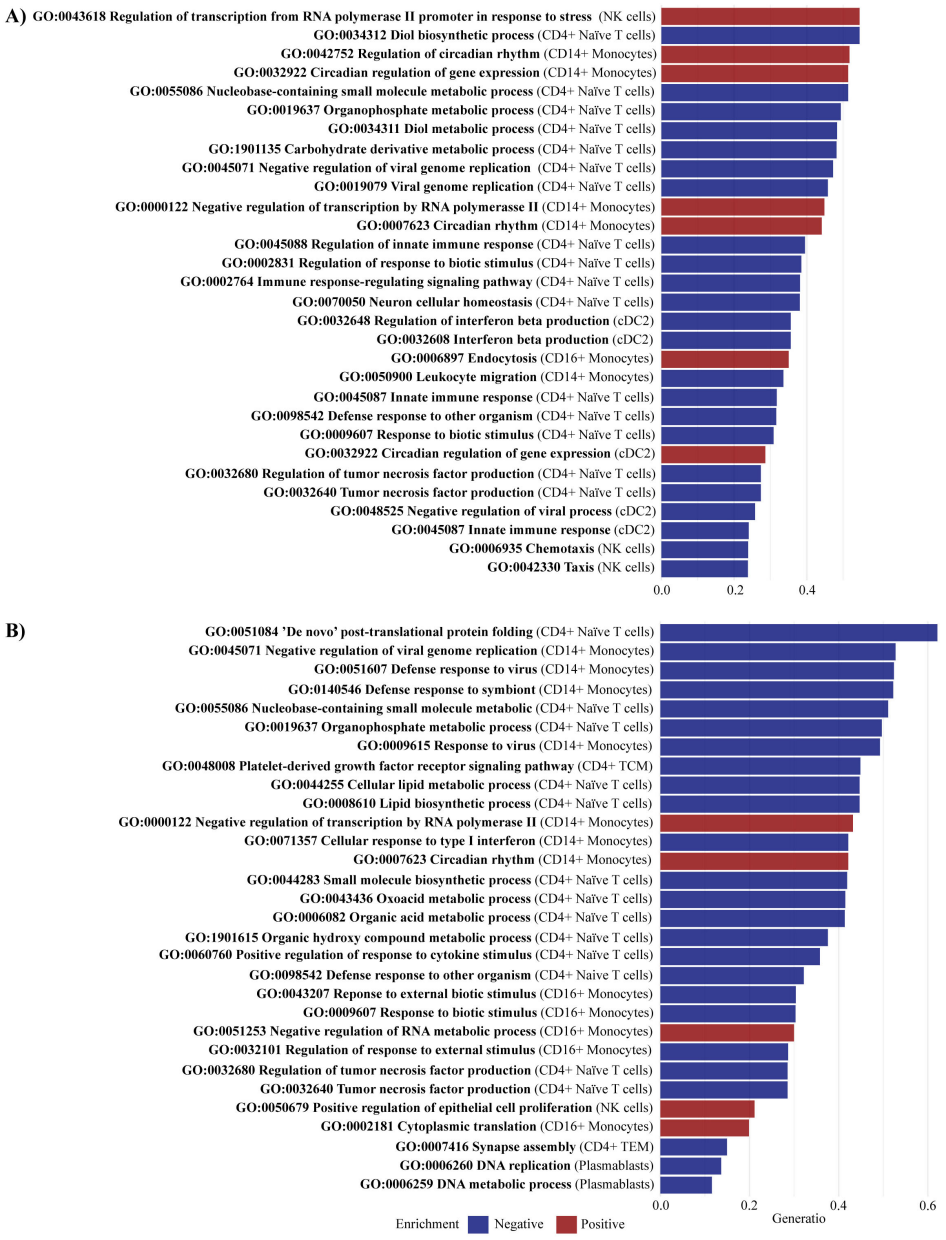
Enriched pathways comparing PsA-only and PsA/PsC vs. healthy controls. Gene set enrichment analysis identified **(A)** for PsA-only vs. healthy controls a total of 75 significantly enriched pathways (adjusted p-value < 0.05), including 37 negatively enriched pathways and 38 positively enriched pathways, and **(B)** for PsA/PsC vs. healthy controls a total of 65 significantly enriched pathways (adjusted p-value < 0.05), including 45 negatively enriched pathways and 20 positively enriched pathways. The figures visualize the top 30 enriched pathways by gene set ratio, describing the overlap between the identified DEGs, and the reference list of genes in the pathway. All pathways are reported in **Supplementary Table S5** and **Supplementary Table S6**. PsA, psoriatic arthritis, PsC, cutaneous psoriasis; NK, natural killer; cDC2, conventional dendritic cells 2 (CD1c-positive).

The GSEA for PsA-only compared with healthy controls identified 75 significantly enriched pathways (adjusted p-value < 0.05), with 37 pathways negatively enriched and 38 positively enriched (**Supplementary Table S5**). In CD4+ Naive T cells, several pathways exhibited negative enrichment, including *Defense response to other organism* (GO:0098542), *Immune response-regulating signaling pathway* (GO:0002764), *Regulation of innate immune response* (GO:0045088), *Regulation of tumor necrosis factor production* (GO:0032680), and *Tumor necrosis factor production* (GO:0032640). Additionally, *innate immune response* (GO:0045087) was negatively enriched in CD4+ Naive and cDC2 cells. Significant DEGs in the core enrichment included CD180 and TNFAIP8L2, which were both upregulated (log2fc of 1.22) in PsA-only patients compared with healthy controls (**Figure 6A** **).** In cDC2 cells, negative enrichment was observed in *Regulation of interferon beta production* (GO:0032648) and *Interferon beta production* (GO:0032608). Interferons are a well-known contributor to psoriatic inflammation (28). Furthermore, positive enrichment was observed in pathways related to *epidermis development* (GO:0008544). The core enrichment genes included MAFF, associated with keratinocyte proliferation (29), which was significantly downregulated (log2fc = −1.85) in PsA patients compared with healthy controls. For CD14+ monocytes, pathways involved in *leukocyte migration* (GO:0050900) showed negative enrichment. The core enrichment genes VAV1 and CD99L2 were significantly DEGs and upregulated (log2fc = 0.59 and 0.68, respectively) in PsA patients compared with healthy controls. Other negatively enriched pathways were observed in natural killer (NK) cells, particularly those involved in *chemotaxis* (GO:0006935) and *taxis* (GO:0042330).

GSEA identified 65 significantly enriched pathways (adjusted *p*-value < 0.05) in PsA/PsC patients compared with healthy controls, with 45 pathways showing negative enrichment and 20 showing positive enrichment (**Supplementary Table S6**). In CD4+ naïve T cells, negatively enriched pathways included the *regulation of tumor necrosis factor production* (GO:0032680), *tumor necrosis factor production* (GO:0032640), and *positive regulation of response to cytokine stimulus* (GO:0060760) (**Figure 6B**). The significant DEGs IFIH1 (log2fc = 0.54) and IFNGR1 (log2fc = 1.23) were part of the core enrichment genes for these pathways and were found upregulated in PsA patients. In CD14+ monocytes, the pathway *Cellular response to type I interferon* (GO: 0071357) was negatively enriched, whereas in NK cells, pathways related to the *Positive regulation of epithelial cell proliferation* (GO:0050679) were positively enriched.

## 4 Discussion

This study aimed to explore the clinical heterogeneity of PsA patients, and how the immune cell composition and transcriptional patterns might differ depending on the clinical phenotype, i.e., PsA patients with and without cutaneous involvement. We utilized scRNAseq of PBMC cells from a total of 58 patients and 10 healthy controls, to gain further understanding of the heterogeneity of the immune cells, identification of specific gene signatures, and biological pathways distinguishing PsA-only and PsA/PsC patients from healthy controls. No difference in clinical measures depicting inflammatory arthritic activity, i.e., DAPSA, DAS28CRP, and SPARCC, was found between patient groups, i.e., PsA-only and PsA/PsC patients. The clinical similarities justified a direct comparison of immune response mechanisms in PsA-only and PsA/PsC patients, minimizing bias that might be introduced by inflammatory disease activity, different immunosuppressive treatments, etc.

Differential expression analysis demonstrated DEGs in PsA patients, including both PsA-only and PsA/PsC patients, well-known and associated with T-cell differentiation, and inflammatory arthritides, including EOMES and NCAP genes in several immune cell subtypes, promoting the importance of CD4+ helper T cells (30, 31). Even though up- and downregulation of individual genes might point in different directions of T-cell differentiation, it mirrors the significance of T-cell plasticity in PsA (32, 33) or the importance of different T-cell subsets, including Th1 and Th17 cells (8). The DE analysis further identified several genes associated with inflammation and autoimmune diseases in the top-20 DEGs such as RGS1, which has been associated with several autoimmune diseases (34, 35). RGS1 is essential for T-cell-mediated immunity and possible for the development of inflammation, as RGS1 depletion has been shown to reduce T-cell migration to the inflammatory site (36, 37) in line with the RGS1 downregulation seen in this study. The downregulation of NCAPG2 might be considered divergent in association with PsA immunopathogenesis, as NCAPG2 and additional NCAP genes have been found upregulated in IL-17+ cells compared with IL-17 cells (31). However, downregulation of NCAPG2 has further been associated with response to TNFi (38), which is highly relevant to acknowledge as 86.2% of PsA patients in the current study have been treated with another bDMARD on a previous occasion. Upregulated genes included CX3CR1, which has been considered important to the pathogenesis of arthritis (39), and the CX3CR1-expressing monocytes have been associated with increased arthritic pain (40). Additionally, both CISH and CLU were found upregulated in I) CD4+ TCM and CD4+ TEM and II) CD14+ monocytes, respectively, which are associated with IL-1β-induced and synovial inflammation (41, 42), and increased levels have been related to psoriasis and arthritis (43). Interestingly, five of the top 20 DEGs were found downregulated in MAIT cells, which might both have tissue-protective and pro-inflammatory features (44). Downregulated genes of MAIT cells included CSRNP1, IRS, NR4A2, PMAIP1, and RBM38 of which NR4A2 has been found in psoriatic skin (45). Results further implicate the CD8+ cytotoxic T cells and NK cells, which have gained recognition as possibly important contributors to PsA immunopathogenesis (8, 46). Additional DEGs and top enriched pathways retrieved from the GSEA were associated with especially CD14+ monocytes, which might be associated with the osteoclast differentiation through the upregulation of the TNF receptor superfamily gene member, TNFRSF1A (47–50), and to immune response activation and sustaining inflammation in PsA (51, 52). Results establish the importance of innate factors in PsA immunopathogenesis and PsA as a mixed pattern disease (53).

It was not possible to conduct the GSEA comparing PsA-only patients and PsA/PsC patients as no DEGs were found comparing the two groups directly. This was unexpected considering the number of differentiating DEGs comparing PsA-only and healthy controls versus PsA/PsC patients and healthy controls. These results could be due to differences in the clinical phenotype that are not reflected in the various gene signatures, why it is less likely that the findings can aid in distinguishing PsA with and without cutaneous psoriasis. However, the results of 0 DEGs between PsA-only and PsA/PsC patients may further be caused by low levels of cutaneous psoriasis, i.e., PASI median of 4.45, in the PsA/PsC patient group. Further investigation into differential gene expression in patients with varying degrees of cutaneous psoriasis may benefit from analyses focused on tissue-level cells rather than PBMCs.

Results of the GSEA exploring differences in gene expression comparing PsA patients and healthy controls exposed a significant shift in immune cell distribution supported by positively enriched pathways of leukocyte migration into the extravascular space of PsA patients (GO:0050900, GO:0030335, both in CD14+ monocytes). Evidence of leukocyte activation (GO:0045321, GO:0001775, in CD14+ monocytes) supports the significant role of innate immunity in PsA and psoriasis, possibly contributing to the recruitment of additional immune cells to the inflammatory site (54, 55). Nevertheless, the study underscores the importance of T cells in the immunopathogenesis of PsA (56–58). Interestingly, the relationship between innate and adaptive immunity was notably also elucidated through the suggested interaction between monocytes and B cells (GO:0019724, GO:0016064, both in CD14+ monocytes), which reinforces the growing evidence of the B cells’ role in PsA immunopathogenesis (59). Comparing the GSEA results from the separate analyses of PsA-only patients versus healthy controls and PsA/PsC patients versus healthy controls revealed distinct patterns of pathway enrichment, which suggest differential immune response mechanisms in these patient groups possibly associated with varying degrees of cutaneous involvement. In PsA-only patients, negative enrichment was demonstrated related to *Regulation of interferon beta production* (GO:0032648, cDC2) and *Interferon beta production* (GO:0032608, cDC2), whereas positive enrichment was observed in pathways related to *epidermis development* (GO:0008544) in cDC2, accompanied by downregulation of the MAFF gene. The combination of negatively enriched interferon beta pathways and downregulation of MAFF may potentially influence the absence of PsC development in this group (28, 29). The three pathways were not enriched in PsA/PsC patients. On the contrary, pathways related to the *Positive regulation of epithelial cell proliferation* (GO:0050679) were positively enriched in NK cells, and analysis of PBMCs has previously associated NK cells with increasing PASI score (8). Noticeably, several pathways associated with circadian rhythm (GO:0042752, GO:007623, in CD14+ monocytes) and its regulation of gene expression (GO:0032933 in CD14+ monocytes and cDC2) were positively enriched in PsA-only patients compared with healthy controls, whereas only the pathway circadian rhythm (GO:007623 in CD14+ monocytes) was positively enriched in PsA/PsC patients. This is considered highly relevant to PsA immunopathogenesis as several (pro-)inflammatory cytokines secreted are subjected to circadian variation (60, 61). Interestingly, PsA-only patients experienced statistically significantly higher levels of fatigue compared with PsA/PsC patients. Further investigation into the gene expression associated with fatigue and sleep disturbances in PsA may offer valuable insights into the underlying mechanisms contributing to these symptoms and their relation to inflammatory disease activity.

Limitations to the study included patients with different histories of previous immunosuppressive treatments, including TNFi, IL-17Ai, and MTX, with different effects on the immune response (62, 63). However, baseline characteristics implied no statistically significant difference between groups considering active and previous treatment. It is likely that medical treatment has biased the results evaluating PsA immunopathogenesis and should be taken into account when interpreting the results. This might be the case considering negatively enriched pathways such as *Regulation of tumor necrosis factor production* (GO:0032680) and *Tumor necrosis factor production* (GO:0032640), and the downregulation of the NCAPG2 gene found in the current study, as NCAPG2 overexpression has been associated with inflammation. However, the downregulation has been associated with response to TNFi (38). Longitudinal studies focusing on gene expression over time should be prioritized to gain further insight into the effect of treatment on the immune response and gene expression. Additional limitations include the difference in gender distribution within the PsA-only and PsA/PsC patient groups, which was not controlled for and might cause a gender bias associated with differential gene expression in different sex and sex-related genes, possible technical variations, and differences in sample quality. Technical variation was minimized as it was the same person running all single-cell isolation and cDNA library preparation procedures. Additional variations were captured during the statistical analysis. Some of these factors can be handled during the preprocessing of the data, but the existing methods are not perfect and can eventually “over” or “under” correct the technical variation instead of regressing it (64). Strengths of the study included the level of real-life data from PsA patients treated in clinical practice, the incorporation of three different methods for the identification of DEGs in the differential expression analysis, and the significant DEGs which were selected based on results across all three methods. Moreover, the distribution of the identified cell types was similar across all samples and patient groups minimizing bias associated with analyzing different immune cell subtypes. Lastly, for the GSEA, the gene ranking was conducted by conducting a PCA on the log2 fold changes from all three tools, providing a more reliable ranking, since several methods were included. In summary, we present genes and pathways that distinguish PsA and PsA/PsC patients from healthy controls, as well as the differences between PsA and PsA/PsC patients, contributing to understanding the underlying immune mechanism, which can eventually lead to better treatment decisions of PsA patients.

In conclusion, single-cell transcriptome profiling provided insight into the heterogeneity of PsA patients and revealed specific transcriptome differences that can be used to distinguish subgroups of PsA patients from healthy controls. Since no studies have compared a PsA cohort based on skin symptoms, those findings add to existing knowledge of distinguishing patients within the group and identifying subtypes that can eventually lead to better treatment decisions and understanding of the immunopathogenesis underlying PsA.

## Data Availability

The datasets presented in this article are not readily available because of ethical and privacy restrictions defined by Danish legislation. Request to access the datasets should be directed to bfh-fp- parkerinst@regionh.dk.
