# Supplementary material for: Single-cell RNA sequencing highlights the influence of innate and adaptive immune response mechanisms in psoriatic arthritis
Source: Front Immunol. 2025 Feb 27;15:1490051. doi: 10.3389/fimmu.2024.1490051 (PMC11904337; doi:10.3389/fimmu.2024.1490051)
Supplement: Supplementary file 1 [file Image1.pdf]

Supplementary Material

1 **Supplementary Figure 1.** Patient inclusion and flow

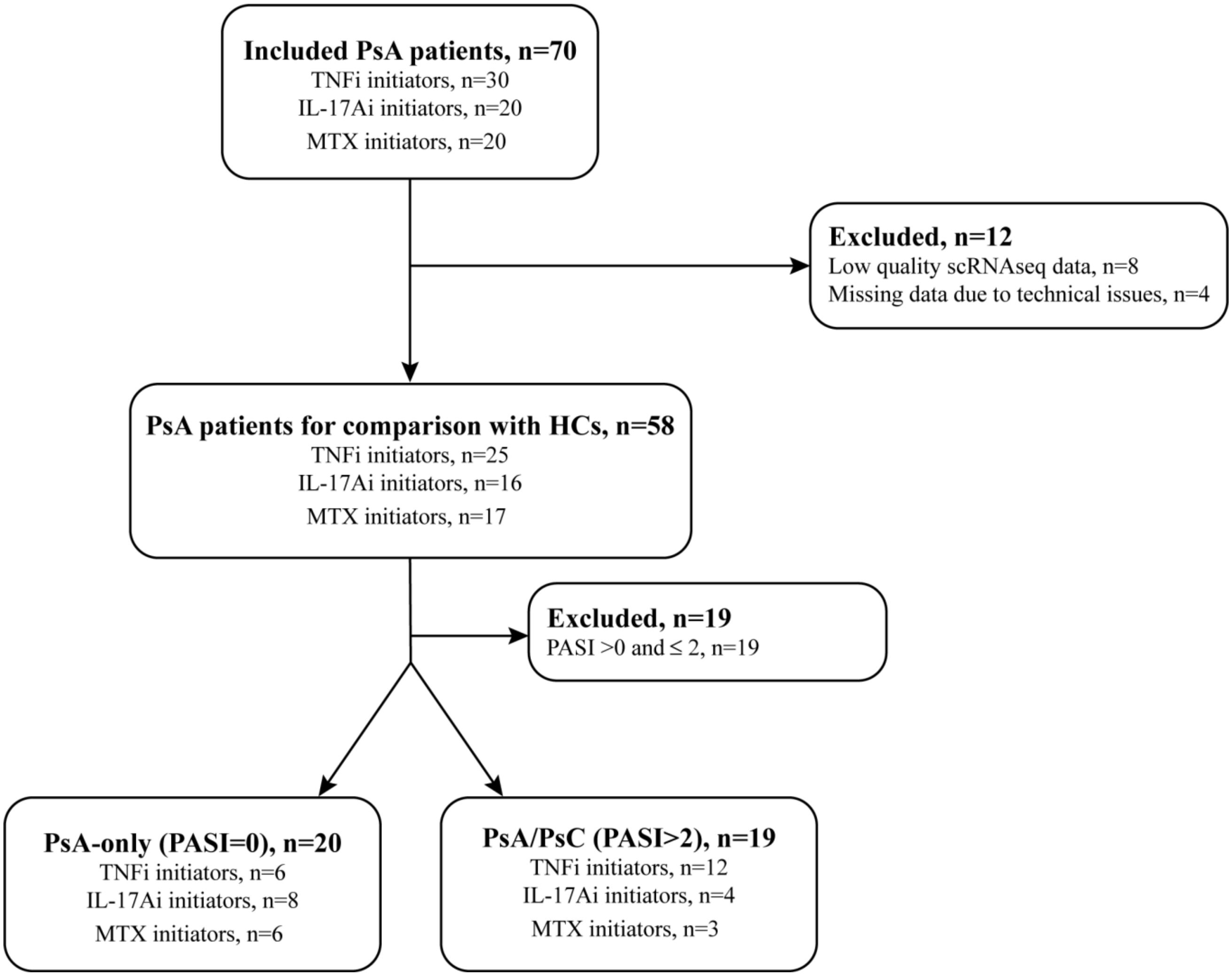

70 PsA patients were included from the Parker Institute's consecutive PsA patient cohort (PIPA). Initial bioinformatical quality control was performed excluding data from 12 patients. Included patients were further stratified based on cutaneous PsC involvement quantified by the PASI score, i.e., PsA/PsC with PASI > 2 and PsA-only with PASI = 0. PsA, psoriatic arthritis; TNFi, tumor necrosis factor alpha inhibitor; IL-17Ai, Interleukin-17A inhibitor; MTX, methotrexate; scRNAseq, single-cell RNA sequencing data; HCs, healthy controls; PASI, psoriatic area severity index; PsC, cutaneous psoriasis

2 **Supplementary figure 2.** Bioinformatical pipeline

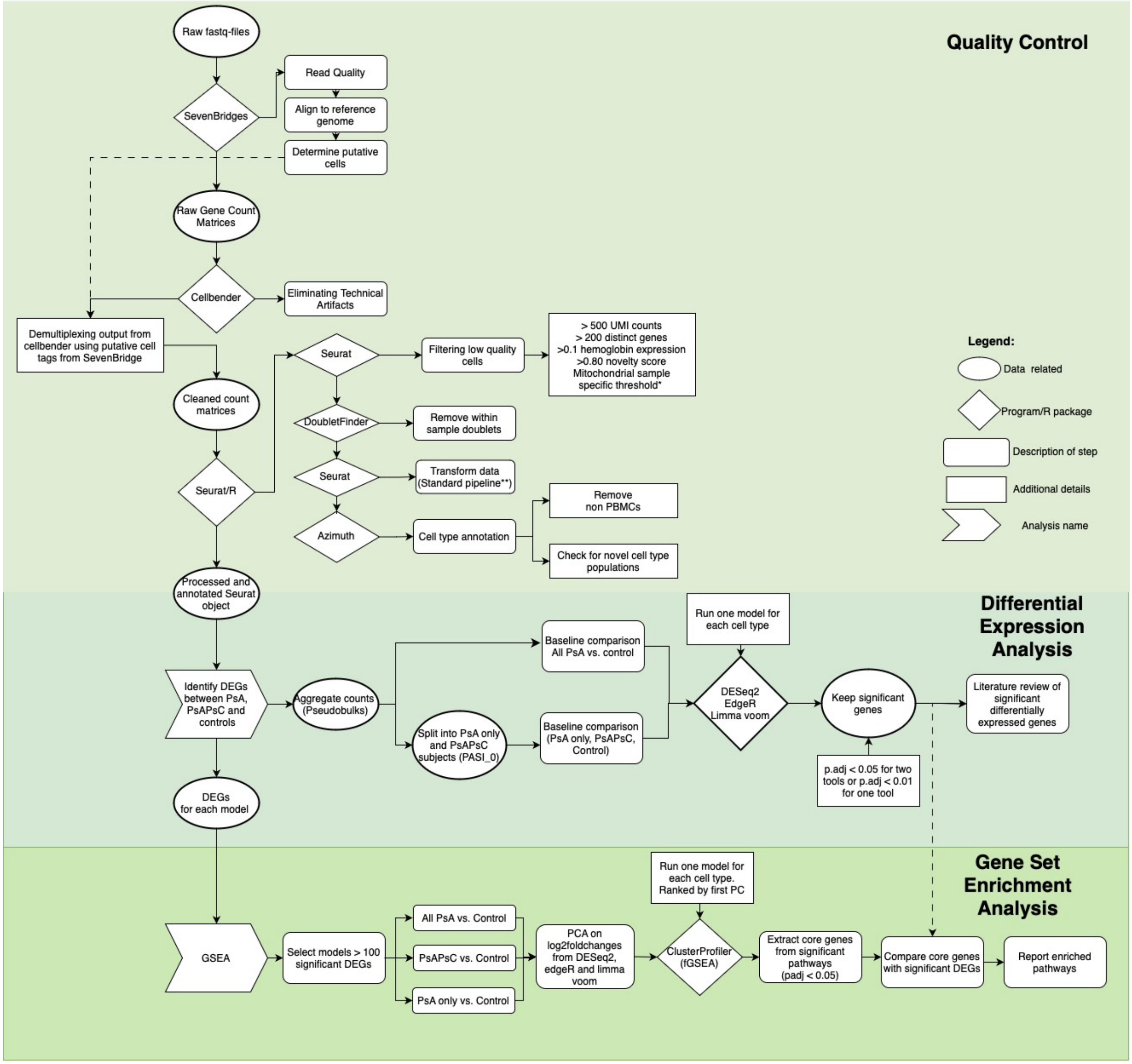

Supplementary Figure 2. An overview of the bioinformatic data analysis performed for the current study. UMI, unique molecular identifier; PBMC, Peripheral blood mononuclear cells; DEGs, differentially expressed genes; PsA, psoriatic arthritis; PsC, cutaneous psoriasis; PASI, psoriasis area severity index; p.adj, adjusted P-value; GSEA, gene set enrichment analysis; PCA, principal component analysis; PC, principal component. *Mitochondrial sample specific threshold: Details of the sample specific mitochondrial threshold are outline in the method section. **Transform data, standard pipeline: The standard pipeline for single cell processing include running the following functions with default parameters from the Seurat package: NormalizeData, FindVariableFeatures, ScaleData (combined in SCTransform function), RunPCA, FindNeighbors, FindClusters, and RunUMAP.

3 **Supplementary figure 3.** Venn diagrams visualizing overlapping genes

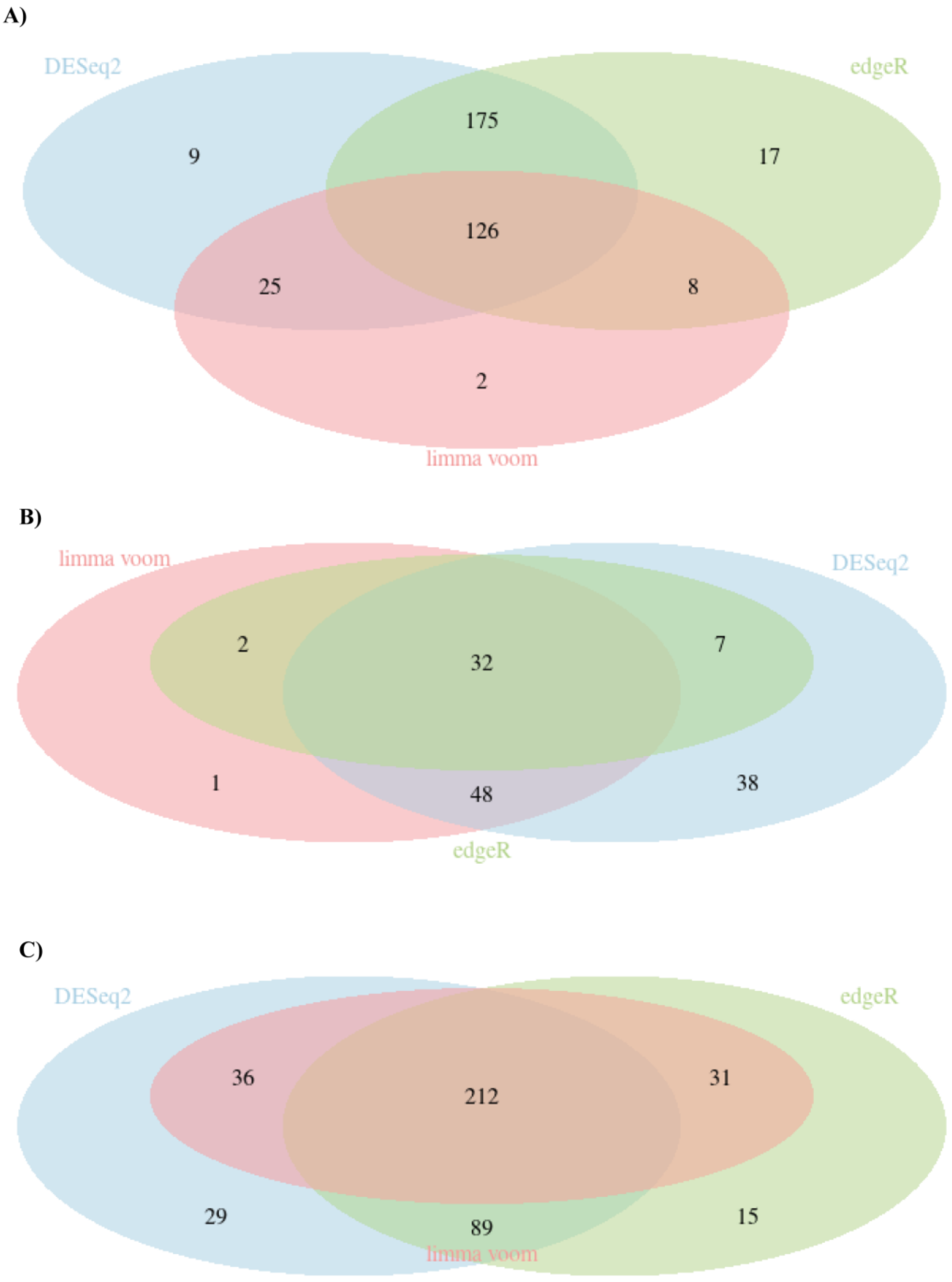

Differential expressed genes were identified using three tools, including DESeq2, EdgeR and Limma-voom. Genes found to have an absolute log2 fold change > 0.5, and found to be significantly differentially expressed (adjusted p-value < 0.05) by at least two of the tools, or found to have an adjusted p-value of < 0.01 by a single tool were considered significantly differentially expressed.

4 Supplementary figure 4. Principal component analysis for gene ranking

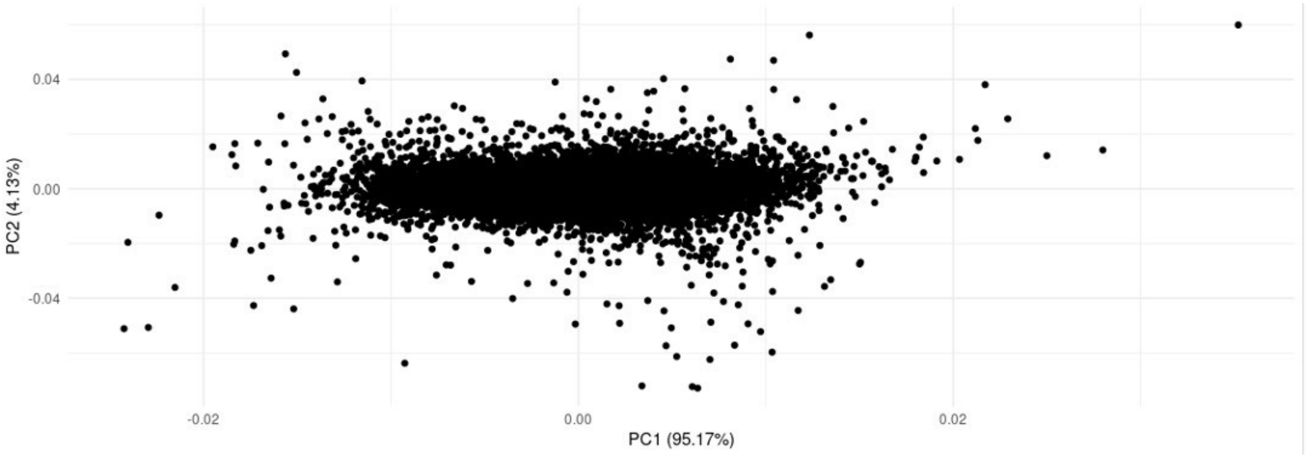

Before the gene set enrichment analysis, a principal component analysis was performed on the log2 fold changes retrieved from DESeq2, EdgeR, and Limma-voom for the PsA-only versus healthy control model. The first principal component was used for gene ranking. PC, principal component.

5 Supplementary figure 5

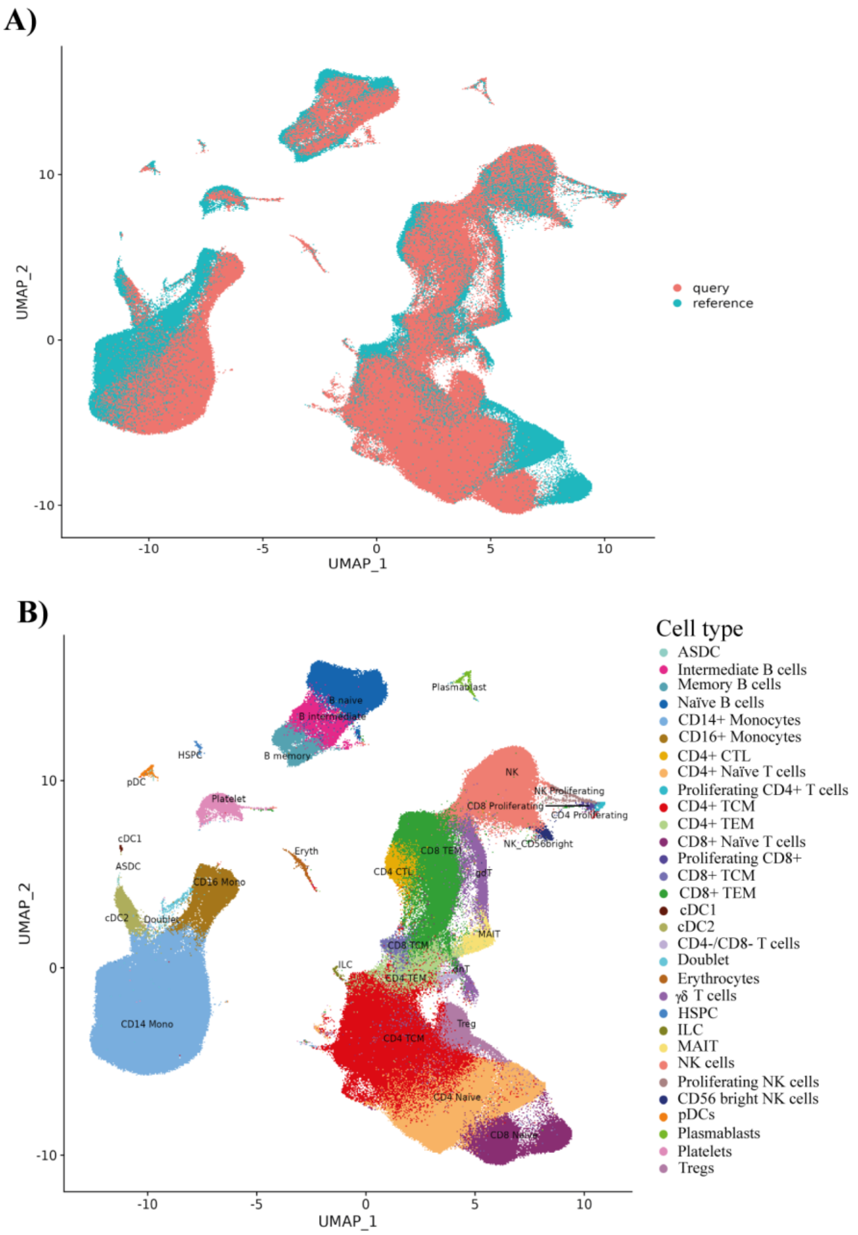

Query cells projected onto reference data set, by calculating a new UMAP embedding. **A)** plot representing cells colored by cell origin (query, or reference) and **B)** plot representing cells colored according to annotated cell type. No novel clusters are identified in the upper plot, indicating that all cell profiles are present in the annotation reference. CD, cluster of differentiation; TCM, central memory T cells; TEM, effector memory T cells; NK, natural killer; MAIT, mucosal associated invariant T cells; Tregs, regulatory T cells; cDC2, conventional dendritic cells 2 (CD1c-positive); pDCs, plasmacytoid dendritic cells; HSPC, hematopoietic stem and progenitor cells; cDC1, conventional dendritic cells 1 (CD141-positive); ILC, innate lymphoid cells; ASDC, AXL+ dendritic cells; CTL, cytotoxic T cells

6 **Supplementary figure 6.** Cell type abundance across included samples

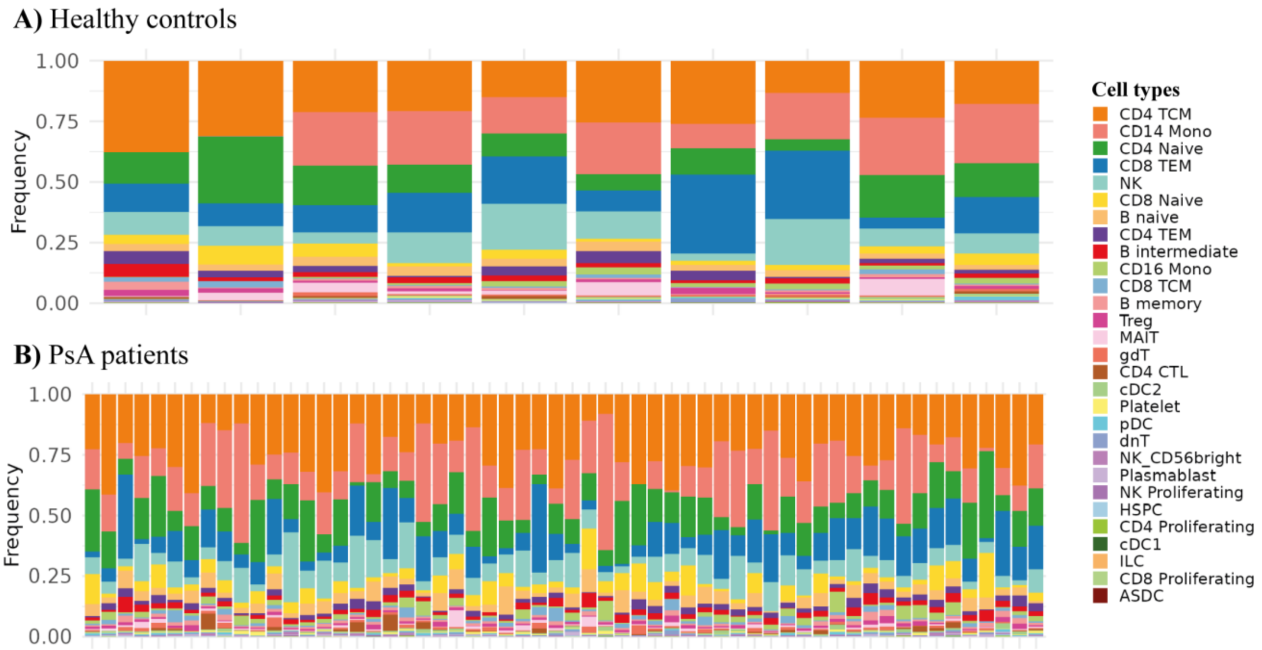

Cell type abundance of different cell types across samples from included patients with one bar representing the frequency of cells in the individual patient. A) represent cell type frequencies from healthy controls and B) represent cell type frequencies from PsA patients. PsA, psoriatic arthritis; CD, cluster of differentiation; TCM, central memory T cells; TEM, effector memory T cells; NK, natural killer; MAIT, mucosal associated invariant T cells; Tregs, regulatory T cells; cDC2, conventional dendritic cells 2 (CD1c-positive); pDCs, plasmacytoid dendritic cells; HSPC, hematopoietic stem and progenitor cells; cDC1, conventional dendritic cells 1 (CD141-positive); ILC, innate lymphoid cells; ASDC, AXL+ dendritic cells; CTL, cytotoxic T cells.

7 **Supplementary figure 7.** Principal component analysis with cell type fractions

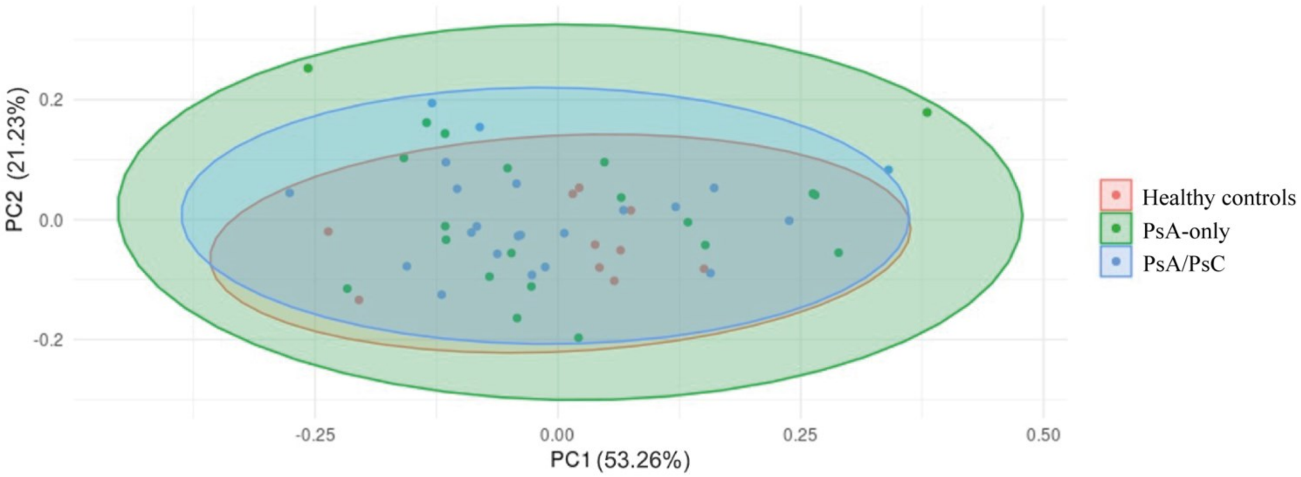

A principal component analysis was conducted on proportions of each cell type within each sample, to explore if the samples would cluster according to difference in cell type distributions. The circles represent the overlap of the three groups. PC1, principal component 1; PC2, principal component 2; PCA, principal component analysis; PsA, psoriatic arthritis; PsC, cutaneous psoriasis

8 **Supplementary figure 8.** Vulcano plots

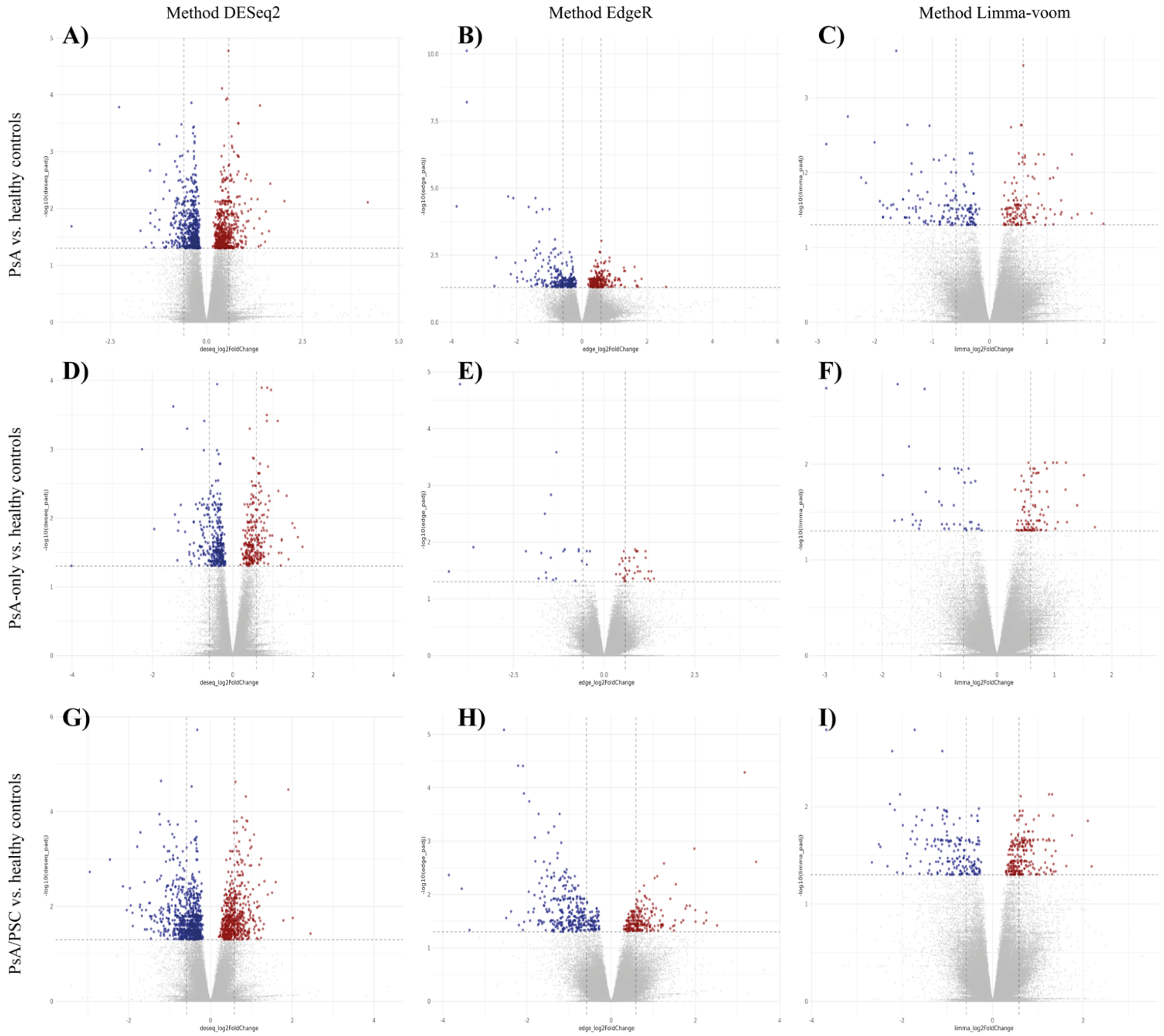

Differentially expressed genes (DEGs) were identified comparing **A-C)** all PsA patients and healthy controls, **D-F)** PsA-only patients and healthy controls, **G-I)** PsA/PsC and healthy controls using three different tools, including DESeq2, EdgeR, and Limma-voom. 298, 113 and unique 308 DEGs were identified comparing all PsA patients and healthy controls, PsA-only patients and healthy controls, and PsA/PsC and healthy controls, respectively. The dashed lines indicates log2 fold change = 0.5, and log10 (p-adjusted = 0.05). PsA, psoriatic arthritis; PsC, cutaneous psoriasis
